# Association between Chronic Misophonia-Induced Stress and Gastrointestinal Pathology in Children—A Hypothesis

**DOI:** 10.3390/children11060699

**Published:** 2024-06-07

**Authors:** Cristina Raluca Bodo, Andreea Salcudean, Aurel Nirestean, Emese Lukacs, Maria Melania Lica, Daniela Lucia Muntean, Ramona Camelia Anculia, Ramona Amina Popovici, Oana Neda Stepan, Virgil Radu Enătescu, Elena Gabriela Strete

**Affiliations:** 1 Department of Ethics and Social Sciences, George Emil Palade University of Medicine and Pharmacy, Sciences and Technology of Târgu Mureș, 540136 Târgu Mureș, Romania; cristina.bodo@umfst.ro (C.R.B.); melaniacozma76@gmail.com (M.M.L.); 2 Department of Psychiatry, George Emil Palade University of Medicine and Pharmacy, Sciences and Technology of Târgu Mureș, 540136 Târgu Mureș, Romania; aurel.nirestean@umfst.ro (A.N.); emese.lukacs@umfst.ro (E.L.); elena.buicu@umfst.ro (E.G.S.); 3 Department of Analytical Chemistry and Drug Analysis, Faculty of Pharmacy, George Emil Palade University of Medicine and Pharmacy, Sciences and Technology of Târgu Mureș, 540136 Târgu Mureș, Romania; daniela.muntean@umfst.ro; 4 Victor Babes University of Medicine and Pharmacy, 300041 Timisoara, Romania; ramonaanculia@gmail.com; 5 Department of Dental Preventive Medicine, Victor Babes University of Medicine and Pharmacy, 300041 Timisoara, Romania; ramona.popovici@umft.ro; 6 Department VIII-Neurosciences, Discipline of Psychiatry, Victor Babes University of Medicine and Pharmacy, 300041 Timisoara, Romania; oana.neda-stepan@umft.ro (O.N.S.); enatescu.virgil@umft.ro (V.R.E.)

**Keywords:** misophonia, pediatric, chronic stress, gastrointestinal disorders, interdisciplinarity

## Abstract

Misophonia is a neurophysiological disorder with behavioral implications, is complex and multifactorial in origin, and is characterized by an atypical and disproportionate emotional response to specific sounds or associated visual stimuli. Triggers include human-generated sounds, mainly sounds related to feeding and breathing processes, and repetitive mechanical sounds. In response to the triggering stimulus, the patient experiences immediate, high-intensity, disproportionate physical and emotional reactions that affect their quality of life and social functioning. The symptoms of misophonia can occur at any age, but onset in childhood or adolescence is most common. Affected children live in a constant state of anxiety, suffer continuous physical and emotional discomfort, and are thus exposed to significant chronic stress. Chronic stress, especially during childhood, has consequences on the main biological systems through the dysregulation of the hypothalamic–pituitary–adrenal axis, including the gastrointestinal tract. Here, we provide arguments for a positive correlation between misophonic pathology and gastrointestinal symptoms, and this hypothesis may be the starting point for further longitudinal studies that could investigate the correlations between these childhood vulnerabilities caused by misophonia and their effect on the gastrointestinal system. Further research to study this hypothesis is essential to ensure correct and timely diagnosis and optimal psychological and pharmacological support.

## 1. Introduction

Misophonia is a neurophysiological disorder with behavioral implications, is complex and multifactorial in origin, and is characterized by an atypical and disproportionate emotional response to specific stimuli [1,2]. Misophonia was described in the early 2000s and has continuously gained clinical and scientific recognition in various medical specialties, yet it is not yet distinctly defined in the Diagnostic and Statistical Manual of Mental Disorders (DSM V) as a psychiatric disorder [2,3].

In response to the triggering stimuli, which can be very diverse, such as human-generated sounds, sounds related to the eating and breathing processes, repetitive mechanical sounds, or foot movement; or visual stimuli normally associated with the auditory trigger—for example, watching people eat—the patient experiences immediate, high-intensity, disproportionate physical and emotional reactions [1,2,3]. This reaction that begins with irritation or disgust immediately escalates to overwhelming experiences of anger and panic [4,5,6]. The extreme, irrational reaction is even associated with feelings of hatred and rage. The reaction is involuntary, with affected individuals stating that they have no control over it, despite understanding its inappropriateness. Moreover, affected individuals have difficulty in distracting their attention from the stimulus [2]. Misophonia is considered a conditioned response to the triggering stimulus [7].

Although the expression of symptoms varies, with mild to severe impairments, misophonia syndrome has a major impact on the quality of life of patients, who develop avoidant, maladaptive behavior, affecting their social life and academic and professional performance [8,9].

Misophonia has been addressed both as an auditory disorder and as a psychiatric disorder. One of the reasons considered for the reluctance to define misophonia as a psychiatric disorder has been the risk of the stigmatization of those suffering from this disorder [10]. However, misophonia cannot be classified as a hearing disorder either because, to date, no association has been found between any hearing impairment and misophonia [10].

Research on misophonia has been growing in recent years, with the first consensus on its definition published in 2022 and presented by Swedo et al. (2022) [2]. This consensus represents a step forward in the acceptance of this pathology as a distinct pathology, with clearly established definition criteria. Due to its valuable contribution in the approach of this pathology diagnosis, we present here the main proposed criteria for misophonia, as follows:

Definition of misophonia: a disorder of impaired tolerance to specific sounds or stimuli known as “triggers”, which are experienced as unpleasant and distressing and evoke negative responses on the emotional, physiological, and behavioral levels; these are responses not seen in other people.

Misophonic response: responses are not corelated with the auditory stimuli intensity, but with a specific pattern or meaning for the affected person.

Misophonic triggers: Triggering stimuli are repetitive and primarily include those produced by humans [2]. Once a trigger is detected, people with misophonia cannot distract their attention from the stimulus. People are aware that their reactions are out of proportion. The symptoms of misophonia usually appear in childhood or early adolescence [2]. Auditory triggers are the most common and are represented by sounds associated with the oral/nasal area including eating, chewing, lip smacking, coughing, gagging, swallowing, breathing, sniffing, and also non-oral/nasal sounds such as clicking, typing, tapping, shuffling, footsteps, clock ticking [2]. Watching a person eat has also been reported as a visual trigger [2].

Misophonic reaction: Reactions to the specific stimulus are defined as the negative affective reactions of anger, irritation, disgust, anxiety, and rage. An arousal of the autonomic system causing muscle tension, increased heart rate and sweating, strong behavioral reactions, agitation, and aggression towards the stimulant takes place. Aggression expressed as verbal or physical outbursts is seen more in children than in adults [2].

Coping mechanism: affected individuals may exhibit behaviors designed to mitigate their reactions such as avoiding situations that could lead to exposure, escaping from triggering stimuli, attempting to interrupt triggering stimuli, and reproducing triggering stimuli [2].

Factors: The factors that determine the intensity of the reaction are linked to the context in which the trigger is encountered; the degree of control perceived by the person over the sound’s source; and, very importantly, the interpersonal relationship with that source. Self-generated stimuli do not usually evoke aversive responses [2].

Impairment: Functional impairment is caused by significant distress that interferes with daily life, leading to mental health problems. Functional impairments span from mild to severe, consisting of difficulty concentrating, an inability to perform professional tasks, and social rejection, leading to impaired functioning in roles, strained social relationships, and social isolation [2].

Association with other comorbidities: misophonia can occur in people having or lacking normal hearing thresholds, in conjunction or not with tinnitus, hyperacusis, neurological or psychiatric conditions such as affective disorders, obsessive compulsive disorders, personality disorders, attention deficit hyperactivity, and autism spectrum disorders [2].

To make the diagnosis of misophonia, its symptoms should not be better explained by any other concomitant disorder [2].

## 2. Etiology of Misophonic Syndrome

The etiology and long-term course of misophonia are not fully known at present. As etiological hypotheses, the existence of traumatic events, a genetic predisposition, and the comorbidity of depression and anxiety have been proposed [1].

Most often, an acute and sudden first appearance is described in childhood or early adolescence, may follow an early triggering event, and may be associated with distressing childhood memories of sounds made by family members, especially during meals. First, the affected person notices sounds made by other people and becomes hypersensitive to them [1,11].

### 2.1. The Genetic Predisposition Hypothesis with Misophonia

The family focus, both in terms of the family as an environmental factor and as a provider of genetic material, is that in over 50% of diagnosed cases, misophonia was discovered in at least one other member of the patient’s family. Studies showing families with different members who meet the diagnostic criteria, or have a family history of the same symptomatology, bring into question the role of the genetic background as well as the role of upbringing and learned behavior within the family [12]. Although there are no twin studies for this pathology, and no genetic studies have been performed to identify the hereditary component [1], some data suggest a substantial impact of genetic factors [13].

The genetic predisposition hypothesis stems from the observation that obsessive compulsive personality disorder, a genetically predisposed disorder, also predisposes individuals to the development of misophonia, and explains the 50% comorbidity rate between the two disorders [11].

There is still little knowledge of how prevalence may differ between biological sexes, but most studies shows that gender is not significantly associated with the misophonic syndrome severity [1,14,15].

### 2.2. The Hypothesis of Neurobiological Alterations

The hypothesis of neurobiological alterations examines the abnormal emotional response of misophonic patients exposed to the trigger from the perspective of the auditory processing mechanism, which might be different in these patients. The association of the inappropriate reaction with a single type of sound suggests that this may be due to a potential anatomical alteration, which could be in the cortical areas responsible for information processing [1], or the limbic and autonomic nervous systems [1], or as a result of hyperconnectivity between the auditory and limbic systems [16]. Somatic symptoms such as high blood pressure and heart rate, physical pain, sweating, and shortness of breath following the exposure to a triggering stimulus are evidence of autonomic nervous system activation [17].

The increased physiological response produces significant distress in affected patients [8] and has a positive correlation with the intensity of patient-reported aversion, suggesting that individuals with misophonia have the same type of reaction to aversive stimuli as the control group, but experience more intense discomfort [17].

Schröder et al. also showed that audiovisual stimuli elicit reactions that occur during exposure to hostile stimuli, which, in addition to affective reactions of anger, disgust, and sadness, also lead to physiological activation with increased activity in the right insula, right superior temporal cortex, and right anterior cingulate cortex, thus demonstrating that they have an exaggerated response to specific stimuli but without permanent functional impairment [18,19].

Another study presented deviant neural activation in the auditory processing system, although the EEG methodology used only allowed for recordings of activity in the cortical areas, having limited anatomical specificity, whereas the misophonic response is likely to involve multiple limbic structures [20,21].

A motor perspective was presented by Kumar et al., who considered that the mirror neuron system linked to orofacial movements and the auditory and visual cortex is at the foundation of this disorder. According to this author, in this pathology, increased connectivity between the orofacial motor areas and auditory cortex in response to all types of sounds may be observed [21].

Because misophonia is not yet accepted as a distinct psychiatric disorder, identifying the neurobiological mechanisms involved in its development could help consolidate misophonia as a distinct pathology and as a psychiatric syndrome in its own right. To date, however, the neurobiological mechanisms of misophonia have been explored very little [19].

### 2.3. The Behavioral Theory of Misophonia

In addition to the genetic predisposition hypothesis and the neurological mechanism hypothesis, behavioral mode hypotheses have also been proposed.

Siepsiak et al. (2023) showed that misophonia in pediatric patients is related to increased psychopathology, but a link between misophonic syndrome and lower social and emotional skills, autism spectrum disorders, and attention deficit hyperactivity disorder cannot be shown. Also, these authors offered a characterization of misophonia as an internalizing rather than an externalizing disorder [13].

A possible positive association with autism spectrum disorders requires further analysis considering that a higher prevalence of misophonic symptomatology was found among patients with autism spectrum disorders [22] and autistic traits were found to be elevated in misophonic patients compared to controls [23]. In a large online clinical sample of adult misophonic patients [5], 3% were diagnosed with comorbid autism spectrum disorder, and 3.6% with self-reported misophonia were diagnosed with autism [24], a prevalence nearly double that of the general population [25,26]. On the contrary, other studies showed no link between misophonic syndrome and autism spectrum disorders [13].

The best known and most accepted behavioral theory of the etiology of misophonia is that it develops through a process of conditioning through an experience, in which a reflex reaction to a predictable stimulus is acquired and repeated, resulting in the association of a stimulus with a negative emotional state.

Thus, misophonia is thought to involve a conditioned response provoked by human audio-visual triggers, with symptoms resulting from increased reactivity in the context of the hypervigilance and sensitization of the auditory cortex [19].

One expert opinion presents several psychological profiles of children who develop misophonia, dividing them into two categories. Both categories are described as having specific symptoms in the feeding process (closely related to meals), which is the primary process of the pathology (most of the triggering sounds are made by the oral area associated with the chewing process). The first category is represented by the obedient and sensitive child. This child is caring, cooperative, and emotional and may not show outward signs of discomfort, but when family members show discomfort, this discomfort is associated with sound. Once the child develops misophonia, he or she becomes demanding of the stimulus and will have specific emotional outbursts. The second category is the strong-willed, volatile child who often has conflicts with a parent or sibling, associating the anger and discomfort felt with the sound, which later becomes the triggering stimulus [27].

We conclude by saying that, at this time, there is no consensus regarding the etiology of misophonia and the mechanisms of the occurrence of its maladaptive behaviors; the authors consider that the lack of generally accepted diagnostic criteria has been the main limitation of the studies conducted with this purpose [28].

## 3. Childhood Clinical Presentation of Misophonic Syndrome and Assessment of Symptoms

The symptoms of misophonia occur at any age. Retrospective studies conducted on adults with misophonia suggest a childhood onset (45%) or adolescent onset (30%), 15% reported life-long presentation, with only 9% reporting that their symptoms began in adulthood [24,29].

The mean age of onset of misophonia was reported to be 12.5 years [11,30,31], with some children meeting criteria as early as 3 years of age. For very young children, it can be difficult to diagnose and differentiate misophonia from other forms of low sound tolerance, as these children are not able to verbalize their needs and discomfort [13]. One specific case has been reported in a 2-year-old child [14]. This suggests that misophonia symptoms may start earlier than previously thought [13,29,32]. However, little attention has been paid to misophonia and its consequences in children and adolescents, although the condition usually begins in childhood [33].

On the other hand, the average age at which misophonia is diagnosed is 37–39 years [11], which is also when patients receive medical attention for the disorder [34].

Misophonia thus becomes a chronic disorder affecting patients for more than 30 years, mainly because their suffering is not usually properly diagnosed and treated [12].

The primary or most triggering stimulus was a family member for 50% of the children included in a study by Siepsiak et al. (2023). [13].These results support the hypothesis that sounds generated by family members are important triggers. This can be explained by the fact that most children spend most of their time within the family; therefore, the disturbing triggers occur in close proximity to them [13,35,36].

A major study of young patients published in 2023 lists the most frequent triggers of misophonia in children as eating (96%), throat sounds (66%), breathing (84%), and tapping (54%). In response, avoidant behavior, annoyance, irritation, verbal aggression, and an impact on family were generally valid. The severity of misophonia was associated with internalizing symptoms with high rates of comorbidity and externalizing behaviors reported by children [4,37].

The misophonic syndrome in children and adolescents can manifest in various ways and intensities and will not necessarily involve externalized verbal or physical aggression. Therefore, screening for self-harm, somatic symptoms, and other related symptoms, should be an important part of the diagnostic process, especially the patients where these externalized behaviors are not obvious [38,39].

The differences in the clinical misophonia presentation in children have also been described by Siepsiak et al. (2023) [13]. In children with misophonia, migraine headaches and severe headaches were reported more than in the control group. They also reported a higher rate of somatic complaints, this correlation being attributed to greater exposure to stress, weaker emotional regulation skills, or emotional suppression [13].

It has been hypothesized that the emotional distress and psychophysiological arousal in misophonia exceed young children’s abilities to respond to a hostile trigger in socially acceptable ways. Guzick et al. (2023) also described verbal and physical aggression in children and adolescents with misophonia [4]. Verbal and physical aggression is considered to be common in misophonia, particularly in children aged 7–12 years, with only 7% reported in adolescent groups. The adolescents evaluated reported self-harm while exposed to trigger sounds. The conclusion presented is that externalized behaviors related to stimuli decrease with age, probably due to increased self-awareness and inhibitory control [4].

The overwhelming majority of sufferers reported that their symptoms became progressively worse with age. The natural course of the disorder is symptom worsening in 45–58% of cases, stable evolution (25%), and, rarely, spontaneous remission (16.7%) [12,17]. With age, misophonia-related distress does not decrease, it is only managed in a better, more socially acceptable manner, but in a self-destructive, dysfunctional way [4]. There is limited research on misophonia across the lifespan, especially among children and adolescents [4].

A milestone in understanding pediatric misophonia is a proper diagnosis, the accurate measurement of symptoms, and an assessment of their impact on daily life and mental and social health [33]. Even for patients at an early age, for the pediatric population, there are some solid psychometric assessment tools using misophonia measurement scales, as shown in Table 1.

## 4. Somatic Implications in Children with Misophonia

Misophonia in children is a vulnerability factor that induces a chronic state of stress that will contribute to later somatic health problems. Children who have misophonia, due to their high level of anxiety, are focused on identifying and listening to any triggering stimulus and avoiding situations, people, and foods that they anticipate will cause discomfort associated with the unpleasant stimulus [17]. Patients thus suffer ongoing physical and emotional discomfort and social maladjustment, contributing to chronic stress and a reduced quality of life [17].

Medical health problems arising from the internalizing process can be affective, including generalized anxiety, panic attacks, and depressed mood; cognitive, including difficulty concentrating and over-focusing on triggers; obsessive, including prevalent or even paranoid ideation about guilt towards themselves and others, as well as avoidance behaviors and coping mechanisms [42,43,44]; and somatic, among which digestive tract disorders are relevant [42,43].

Very few studies have examined somatic effects in misophonic pathology, effects produced by continuous stress, high anxiety, and difficulties with eating properly. A study published in 2022 investigated the possible complications of this pathology and comorbidities in the pediatric population.

A self-assessment questionnaire was used to evaluate a range of medical health problems in children with misophonia and their families, including neurodevelopmental impairment, neurocognitive disorders, gastrointestinal disorders, sensory processing difficulties, and heart and kidney conditions [45]. Acid reflux, migraines, and auditory pathology such as tinnitus and hyperacusis had a significantly positive association with misophonia severity [45]. In an observational study conducted in 2013–2017 on adult subjects with misophonic symptoms, somatic examination results (general screening and hearing tests) showed that irritable bowel syndrome was among the most common somatic diagnoses, along with migraine, asthma, and back pain. During physical examination, no neurological disorders were determined, but mild comorbid somatic disorders were regularly found [5].

## 5. Consequences of Chronic Stress Exposure in Misophonia

The medical term stress, first defined by endocrinologist Hans Hugo Bruno Selye, represents the adaptive response of organisms to adverse endogenous or exogenous, psychological or physical, and real or perceived stressors [46].

### 5.1. Stress and the Hypothalamic–Pituitary–Adrenal Axis

The hypothalamic–pituitary–adrenal axis is the efferent stress axis that interrelates the adaptive responses of the human body to stressors and is a part of the limbic system, the area predominantly involved in emotional responses and memory [47].

The acute stress response is modulated by physiological alterations in the nervous and endocrine system, but eventually, the equilibrium returns to the basal state. On the other hand, chronic stress, especially during childhood, leads to the long-term activation of the stress pathways, and has detrimental psychological consequences for all major biological systems, including the cardiovascular, nervous, endocrine, metabolic, and immune systems [48,49]. Numerous studies have approached the mechanisms connecting early life stress to these systems and have analyzed the relationship between the gut microbiome and stress, specifically in early life [50,51,52].

Chronic psychological stress is considered to be associated with the deregulation of the hypothalamic–pituitary–adrenal axis. Environmental stress, such as that induced by misophonia, activates the system through the secretion of corticotropin-releasing factor from the hypothalamus, followed by the secretion of adrenocorticotropic hormone from the pituitary gland, which will lead to the release of cortisol from the adrenal glands. Cortisol, the main stress hormone, affects all body structures via the blood circulation, allowing the brain to influence the activities of somatic systems including the gastrointestinal system [53].

Also, corticotropin-releasing factor receptors and their ligands can be modulated by stress and are expressed in both the gut and the brain [54], can influence the secretion of the mucosal barrier and enteric peristalsis, and plays an important role in gastrointestinal disorders [55,56,57].

It is known that the gut–brain axis involves two-way communication, joining the central nervous system and the enteric system, and connecting the emotional and cognitive brain areas to peripheral gut functioning [53]. There is clinical evidence of gut–brain axis interactions resulting from the association of dysbiosis with central nervous disorders, particularly in autism spectrum disorders, anxiety–depressive disorders, and functional gastrointestinal disorders. One example of this kind of relationship is irritable bowel syndrome [53].

### 5.2. The Microbiome-Specific Signatures

The majority of preclinical studies investigating the microbiota gut–brain axis have been focused on animal model studies and have suggested that the gut microbiome plays a role in the development of the stress response [58]. There are studies that have shown that the neurotransmitter GABA, which is involved in anxiety and depressive disorders, is secreted by Lactobacillus and Bifidobacterium [59]. The probiotic Lactobacillus rhamnosus has been shown to decrease anxiety behaviors in stress tasks and modify the functioning of GABA receptors in mice [60]. The level of tryptophan, the serotonin precursor, plays a role in depression, and can be elevated by Bifidobacterium ingestion [61]. The influence of the brain on the microbiota structure and function by altering intestinal permeability has also been shown in laboratory animals by allowing bacterial antigens to infiltrate the epithelium and to stimulate the immune response [62].

There is evidence extracted from animal models that suggests that early stress can impact on the long-term stress reactivity and gut microbiome [63] and that exposure to early stressors is associated with a significant decrease in the diversity of the microbiota [64]. Local and general changes in gastrointestinal functionality can influence the delivery of nutrients, prebiotics, and dietary fiber [62].

### 5.3. The Role of Serotonin and Catecholamines

An additional vector of the process is serotonin, with its important role in influencing affect, its involvement in pain control, in the central nervous system, and in the periphery through descending inhibition [65], and its influence on digestion, where it plays an important role in gastrointestinal signaling as a paracrine messenger used by enterochromaffin cells [66].

Stress activates the sympathetic autonomic system, contributing to the increased production of adrenal hormones. Catecholamines mediate the increase in central and peripheral inflammatory cytokines [67]. In addition, the vagus nerve, and its anti-inflammatory effects, are decreased by stress, contributing to an increased inflammatory response and, consequently, gut inflammation [68,69].

**Figure 1:**
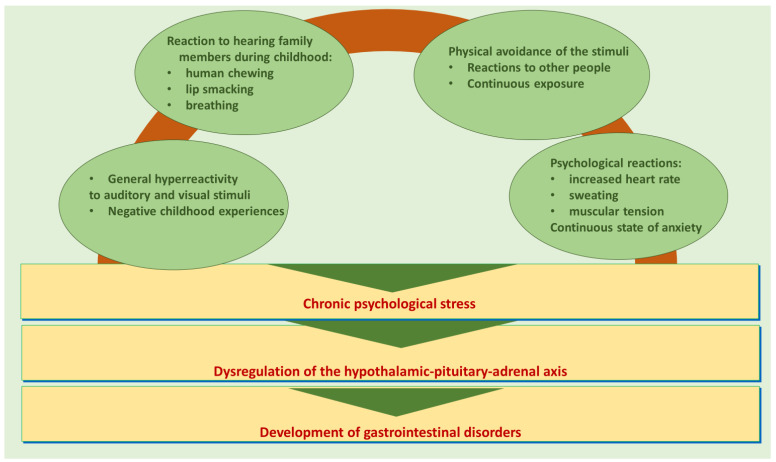
Proposed model for the development of gastrointestinal pathology in misophonic syndrome.

Stress and anxiety, through the mechanism outlined above, can be top triggers for many gastrointestinal pathologies, especially in children [70]. By correlating misophonia-induced stress and its effect on the gastrointestinal system with the mechanisms presented, we propose a model to justify the occurrence of gastrointestinal pathology in the pediatric population suffering from misophonia. This is shown in Figure 1.

## 6. Therapeutic Approach

Although our understanding of misophonia is increasing, and the associated chronic distress is being accepted more by clinicians, there is limited knowledge about effective treatments that can be offered to patients [5,31,71,72].

As the field advances and a better understanding of the pathology is gained, more treatment options will become available, but it is not yet known which therapies are the most effective or which are acceptable for parents and their children with misophonia and associated somatic distress.

Understanding patient preferences has important clinical implications. Patient involvement in the treatment approach, including treatment beliefs and affordability, contributes to treatment acceptance and initiation and clearly leads to better adherence and patient outcomes [73,74]. In the child and adolescent population, understanding the treatment preferences of both the parent and child are equally important, given that the treatment decision is shared equally by the clinician, parent, and child [75,76].

After a thorough study of the adverse effects of stress on digestive pathology, the improvement of the mental status seems to be the main goal of treatment and is expected to even outpace the healing process of the lining of the gastrointestinal system [56,77,78].

Reducing psychological stress is important for symptom alleviation and increasing the quality of life. Thus, the new field of psychogastroenterology focuses on the application of psychotherapy as an integral part of digestive disease management [56,78].

The field of psychogastroenterology has made major progress in identifying psychosocial risk factors for adverse outcomes in people with gastrointestinal disease. These factors include anxiety, depression, and maladaptive behaviors [79]. Based on the biopsychosocial model [80], the integration of psychological approaches into the understanding and treatment of gastrointestinal disorders has become a standard of care. The most commonly applied psychological treatment is cognitive behavioral therapy, with strong evidence of its effectiveness in treating children and adults suffering from irritable bowel syndrome and inflammatory bowel disease [81,82,83]. It may also be proposed in the setting of misophonic pathology.

The effectiveness and mechanism by which psychological treatments work in gastrointestinal disorders are not fully understood. Cognitive behavioral therapy for digestive pathology focuses on reducing psychological distress and increasing the quality of life. There is a growing awareness that psychological trauma impacts gastrointestinal functioning [84].

In a recent systematic review, Glynn et al. [85] identified that the cumulative prevalence of trauma in gastrointestinal cohorts was 36%. Given the influence of trauma on gastrointestinal disease, Jagielski et al. examined whether patients with a history of trauma were more likely to respond to psychological interventions [86]. An optimal effect of psychological interventions has also been identified in other somatic pathologies, such as type 1 diabetes in children [87].

These findings underline the importance of identifying early misophonic pathology, identifying associated somatic distress, and providing them and their parents with well-planned and -executed interventions that can prevent or mitigate potential medical and social effects as well as their chronicity [13].

## 7. Conclusions

Systematic data on the clinical presentation of this pathology in children and adolescents are of paramount importance, as developing an early understanding of the condition is the basis for appropriate treatment.

The early detection of misophonia and effective interventions can mitigate the long-term adverse effects of misophonic syndrome on mental health, somatic health, and social life.

In this paper, we evaluate the hypothesized correlation between misophonia and the chronic stress induced by it in children and its effect on the proper functioning of the digestive tract, attempting to provide a framework for understanding misophonia based on widely accepted physiological processes.

Although the information presented here only consists of preliminary arguments justifying a possible positive correlation between gastrointestinal symptoms such as acid reflux, irritable bowel syndrome, and functional abdominal pain; the pattern of misophonic pathology; the setting of its manifestation; its close relationship with nutrition; and the act of eating, we believe that this hypothesis could be the starting point for further studies that can further elucidate how misophonia affects the somatic well-being of affected children.

However, longitudinal studies are needed to investigate the correct correlations between these early life vulnerabilities such as misophonia, anxiety, and mental health, and their effect on somatic status, including the gastrointestinal system. Further research to validate or invalidate this hypothesis is essential to ensure correct and timely diagnosis and optimal psychological and pharmacological support.

## Figures and Tables

**Table 1:** Summary of validation scales available for pediatric use for misophonia.

Scale	Description
MAQ [33]	The scale assesses the negative effects of misophonia in everyday life, a multidimensional measure with four identical scales, with one version for children and one for parents: interference, pessimism, non-recognition, and current distress. The MAQ can also be used to detect the negative consequences of misophonia.
A-MISO-S [33]	This scale is a unidimensional measure of the overall severity of pediatric misophonia and is based on the Yale–Brown Obsessive Compulsive Disorder Scale. It assesses stress, time, control, interference, resistance, and avoidance.
Misophonia Impact Questionnaire (MIQ) [40]	This is a promising questionnaire for assessing the impact of misophonia in adolescents, but is completed by a parent.
Sussex Misophonia Scale for Adolescents [6]	This scale is a tool for measuring misophonia in adolescents, based on the Sussex Misophonia Scale for Adults. The scale is ideally suited for adaptation in adolescents by using psycholinguistic data to ensure child-appropriate language.
Misophonia Scale Amsterdam—Youth [4,41]	This scale is an adaptation of the adult self-assessment questionnaire AMISO-S and the revised AMISOS-R, but it is specifically adjusted for the particularities of Dutch children.
